# Research Progress of DUB Enzyme in Hepatocellular Carcinoma

**DOI:** 10.3389/fonc.2022.920287

**Published:** 2022-06-27

**Authors:** Jie Zhao, Jinhui Guo, Yanan Wang, Qiancheng Ma, Yu Shi, Feng Cheng, Qiliang Lu, Wen Fu, Guangxiong Ouyang, Ji Zhang, Qiuran Xu, Xiaoge Hu

**Affiliations:** ^1^College of Biotechnology and Bioengineering, Zhejiang University of Technology, Hangzhou, China; ^2^Laboratory of Tumor Molecular Diagnosis and Individualized Medicine of Zhejiang Province, Zhejiang Provincial People’s Hospital, Affiliated People’s Hospital, Hangzhou Medical College, Hangzhou, China; ^3^Qingdao Medical College, Qingdao University, Qingdao, China; ^4^Department of Hepatobiliary and Pancreatic Surgery, Zhejiang Provincial People’s Hospital, Affiliated People’s Hospital, Hangzhou Medical College Hangzhou, China; ^5^The Key Laboratory of Tumor Molecular Diagnosis and Individualized Medicine of Zhejiang Province, Zhejiang Provincial People’s Hospital, Affiliated People’s Hospital, Hangzhou Medical College, Hangzhou, China

**Keywords:** ubiquitin, deubiquitinating enzymes, hepatocellular carcinoma, targeted, structure, inhibitors

## Abstract

According to GLOBOCAN 2021 cancer incidence and mortality statistics compiled by the International Agency for Research on Cancer, hepatocellular carcinoma (HCC) is the most common malignancy in the human liver and one of the leading causes of cancer death worldwide. Although there have been great advances in the treatment of HCC, such as regofenib, sorafenib, and lomvatinib, which have been developed and approved for the clinical treatment of advanced or metastatic HCC. However, they only prolong survival by a few months, and patients with advanced liver cancer are susceptible to tumor invasion metastasis and drug resistance. Ubiquitination modification is a type of post-translational modification of proteins. It can affect the physiological activity of cells by regulating the localization, stability and activity of proteins, such as: gene transcription, DNA damage signaling and other pathways. The reversible process of ubiquitination is called de-ubiquitination: it is the process of re-releasing ubiquitinated substrates with the participation of de-ubiquitinases (DUBs) and other active substances. There is growing evidence that many dysregulations of DUBs are associated with tumorigenesis. Although dysregulation of deuquitinase function is often found in HCC and other cancers, The mechanisms of action of many DUBs in HCC have not been elucidated. In this review, we focused on several deubiquitinases (DUBs) associated with hepatocellular carcinoma, including their structure, function, and relationship to hepatocellular carcinoma. hepatocellular carcinoma was highlighted, as well as the latest research reports. Among them, we focus on the USP family and OTU family which are more studied in the HCC. In addition, we discussed the prospects and significance of targeting DUBs as a new strategy for the treatment of hepatocellular carcinoma. It also briefly summarizes the research progress of some DUB-related small molecule inhibitors and their clinical application significance as a treatment for HCC in the future.

## Introduction

Liver cancer is a common cause of cancer death worldwide and is one of the ten cancers with a high incidence (1). Due to the asymptomatic nature of early hepatocellular carcinoma (HCC), HCC can only be evaluated by some early biomarkers in the patient’s body, such as serum α-fetoprotein (AFP) (2), Glypican-3 (GPC3) (3), and tumor-associated antigens (TAAs) (4). As a result, most patients are unable to detect and treat HCC at an early stage; moreover, HCC has a poor prognosis and a high mortality rate (5). For patients with early and intermediate HCC, surgical therapies such as hepatic resection and liver transplantation have good results (6). However, surgical therapy needs to consider factors such as the patient’s tumor stage and physical condition, so surgical therapy is not suitable for some patients. At present, systemic therapy and some adjuvant therapies of clinical surgery have become new research strategies for the treatment of HCC (6), such as transarterial chemoembolization (TACE), transarterial radioembolization (TARE), external beam radiation therapy, and oncolytic virus (7), but the effect of these treatments is not ideal. Systemic drug therapy has also become an important means of current liver cancer treatment (8). At present, many targeted drugs have been approved for the clinical treatment of HCV patients, for example, Nexavar (sorafenib), an oral drug first approved to target multiple kinases (9); regorafenib (Stivarga) was approved in June 2017 (10); and lenvatinib (11). These drugs all provide new treatment directions for HCC patients.

As we all know, the pathogenesis of human HCC is more complex, and an in-depth understanding of the molecular mechanism of HCC pathogenesis can provide an effective treatment strategy for improving the survival rate of HCC patients. At present, the development of targeted drugs provides new therapeutic prospects for the current treatment of HCC. Signaling pathways and potential targets related to the pathogenesis of HCC have become important methods for the development of drugs targeted for the treatment of advanced HCC (12). Studies have reported many key targets associated with HCC, such as microRNAs (miRNAs) and long non-coding RNAs (lncRNAs) (13), programmed cell death-1 and its ligands (PD-1/PD-L1) (14), hypoxia-inducible factor (HIF) (15), and deubiquitinases (DUBs) (16).

**Figure 1:**
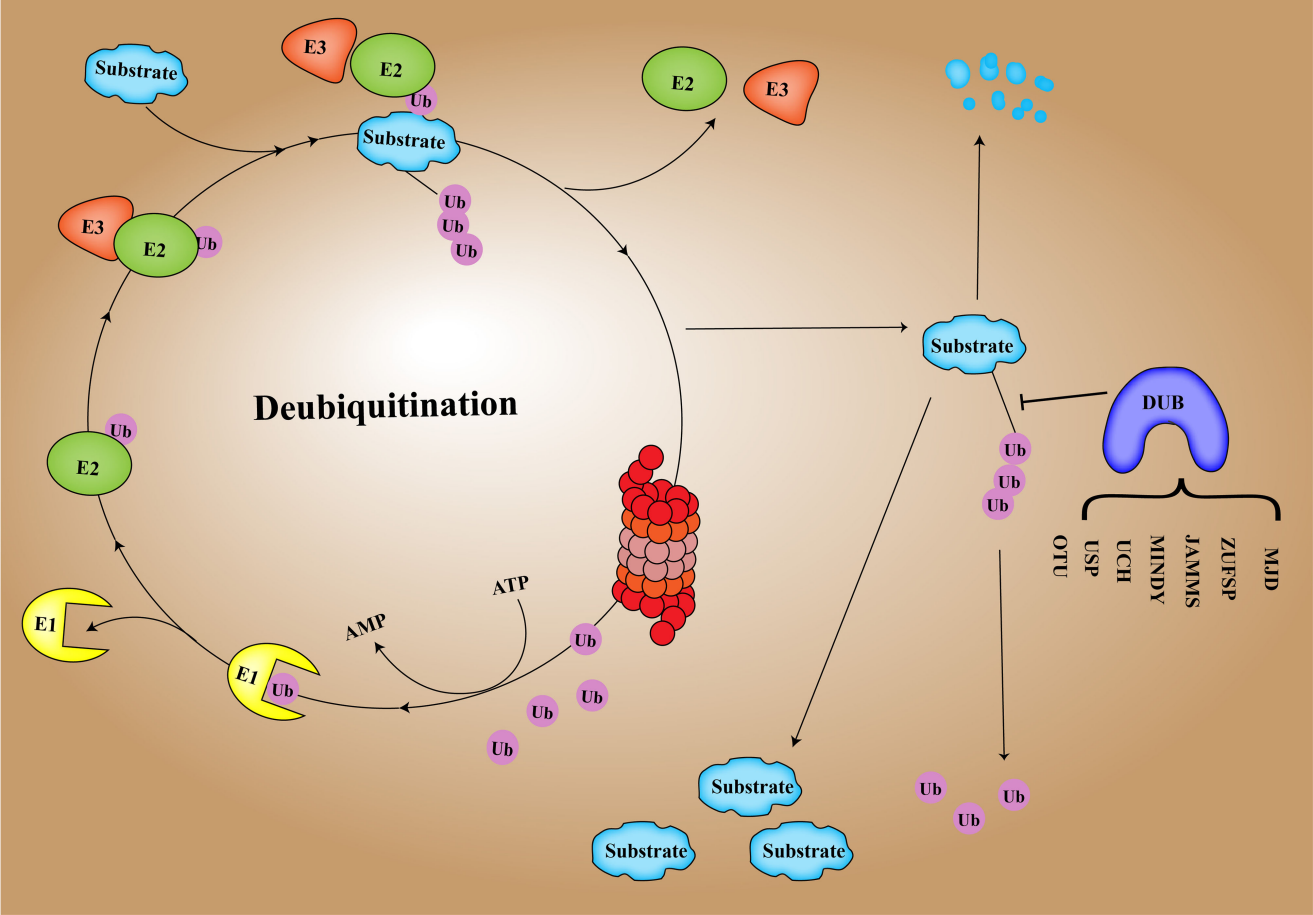
Ubiquitination of E1, E2, E3, and deubiquitination mechanism of DUB.

DUB is an important regulator of the process of deubiquitination and ubiquitination balance in human cells (17). Ubiquitination of proteins is a process in which multiple ubiquitin molecules are covalently attached to the protein substrate and then degraded by the 26S proteasome complex under the combined action of three types of enzymes: ubiquitin-activated enzyme (E1), ubiquitin-coupled enzyme (E2), and ubiquitin ligase (E3) (18, 19). Ubiquitination involves seven lysine residues: K6, K11, K27, K29, K33, K48, and K63 and N-Teline (Met1) (20). These residues can be ubiquitinated to form isopeptide-linked ubiquitin chains (21). DUBs include cysteine proteases as well as metalloproteinases that specifically cleave ubiquitin molecules in protein substrates (22). Regulating the homeostasis of ubiquitination and deubiquitination is conducive to the normal progress of human cell activities and maintains homeostasis in the human body (23). There are approximately 100 DUBs in humans, and DUB enzymes can be divided into 7 families based on structure and function (24), including ubiquitin-specific proteases (USPs), ubiquitin C-terminal hydrolases (UCHs), proteases containing the Machado–Joseph domain (MINDYs), ovarian tumor proteases (OTUs), newly discovered zinc finger protease (ZUPs/ZUFSPs), JAM/MPN domain-related Zn-dependent metalloproteinases (JAMMs), and Machado–Josephin domain-containing proteases (MJDs) (25) (**Figure 1**).

**Figure 2:**
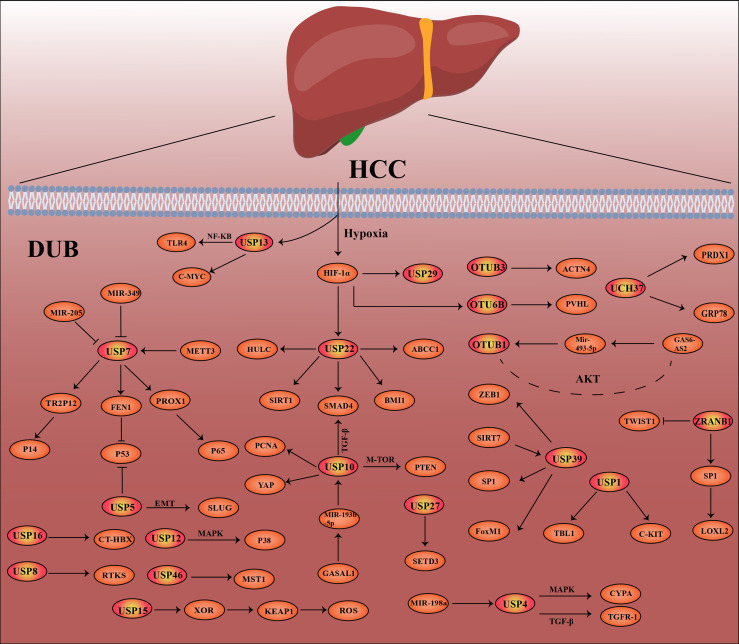
Deubiquitinase-related proteins and pathways in hepatocellular carcinoma (HCC).

At present, a number of studies have shown that the deubiquitination effect of proteins is closely related to the occurrence and development of cancer, such as breast cancer, lung cancer, stomach cancer and hepatocellular carcinoma (26–30). In this review, we highlight hepatocellular carcinoma -related DUBs, including their structure, mechanisms of action in hepatocellular carcinoma, and recent research advances. In **Figure 2**, the related pathways and target proteins of DUBs in HCC are shown (**Figure 2**). Last but not least, we discussed the prospects and implications of DUBs and DUB-related small molecule inhibitors as potential protein targets for hepatocellular carcinoma treatment.

## Ovarian Tumor Protease OTU

There are 16 species of cysteine protease OTU family members, which can be divided into four different subfamilies: OTUB subfamilies (OTUB1 and OTUB2), OTUD subfamilies (OTUD1, OTUD2/YOD1, OTUD3, OTUD4, OTUD5/DUBA, OTUD6A, OTUD6B, and ALG13), A20-like subfamilies (A20, Cezanne, Cezanne2, TRABID, and VCPIP), and OTULIN subfamily (OTULIN) (31). Studies have shown that the Cys catalytic residues present in the OTU subfamily protease active site make it susceptible to reverse oxidation (32). Here, we introduce the structure and function of “OTUB1 OTUD3 OTUD6B ZRANB1” in the OTU family and the research progress in HCC.

### OTUB1

#### Structure of OTUB1

OTUB1 is a founding member of the ovarian tumor (OTU) domain family of DUBs and belongs to the OTUB subfamily (33). In addition to the OTU domain containing 130 amino acids, OTUB1’s unique crystal structure has two different ubiquitin-binding sites (34). During the deubiquitination process, OTUB1 preferentially cleaves the polyubiquitin chains connected by Lys (34, 35) while using the active center to catalyze the substrate reaction. The catalytic domain of OTUB1 consists of three parts: Cys(C)91, His(H)265, and Asp(D)268 (36). Studies have shown that in the presence of free ubiquitin molecules, the activity of the OTUB1 enzyme is regulated by the E2 enzyme: the uncharged E2 enzyme can activate the activity of the OTUB1 enzyme by stabilizing the N-terminal structure of OTUB1. In addition, OTUB1 is able not only to remove the ubiquitin molecules linked to the substrate but also to inhibit the ubiquitination process through binding to the E2 enzyme (37).

#### The Function of OTUB1 and Research Progress in Hepatocellular Carcinoma

OTUB1 is expressed in a variety of tissues in the body, such as the kidneys, colorectum, stomach, brain, and liver (38). In human liver cancer and other tumor tissues, OTUB1 has been shown to have a high expression and is associated with a poor prognosis in patients (38, 39). Inhibiting the expression of OTUB1 by shRNA will weaken the proliferation, migration, and invasion ability of HCC cells (38).

LncRNAs are a class of RNAs that are not protein-coding and can bind to downstream MIR genes through endogenous competition and targeted action (40, 41). It is widely believed to be associated with many diseases in the human body and is also a related causative agent of cancer (42). OTUB1 is also associated with lncRNA in liver cancer. LncRNA GAS6-AS2 was shown to be upregulated in liver cancer cells as well as tissues. GAS6-AS2 regulates the expression of downstream OTUB1 by targeting miR-493-5p with 3′UTR (39). The hyperactivated PI3K/Akt signaling pathway plays a central role in cancer cell metabolism and is also thought to be associated with the occurrence of HCC as well as metastasis (43, 44). LncRNA GAS6-AS2 knockdown can promote HCC cell proliferation, invasion, metastasis, and apoptosis by mediating the miR-493-5p/OTUB1 axis to activate the PI3K/AKT/FoxO3a pathway (39). The above studies show that OTUB1 can be used as a novel marker for targeted therapy for liver cancer.

### OTUD3

#### Structure of OTUD3

OTUD3 belongs to the subfamily OTUD and is structurally similar to OTUD2. Its active domain is approximately 52–209 amino acids, which also includes the DUB family’s classic catalytic triplet residues (31). During the deubiquitination process, OTUD3 is the only DUB that tends to cleave k6-linked double ubiquitin and bind it to the S1 and S1′ sites (31). The Lys6-linked polyUb is a mysterious type of chain whose role in cells has not yet been elucidated (45).

#### The Function of OTUD3 and Research Progress in Hepatocellular Carcinoma

A growing number of reports suggest the role of OTUD3 in human cancers, such as breast cancer as well as lung cancer (35, 46). Studies have demonstrated that OTUD3 is expressed in high amounts in HCC tissues and is associated with a poor prognosis in HCC patients (47). α-Actin 4 (ACTN4) is called an actin-binding protein and belongs to a family of actin-binding proteins. OTUD3 can affect the expression of α-actin 4 (ACTN4) at the protein level and promote the proliferation, invasion, and metastasis of HCC by stabilizing ACTN4 by deubiquitination (47).

## Other Enzymes of the OTU Family

### OTUD6B

Studies have shown that OTUD6B can regulate HCC metastasis by regulating the activity of HIF under hypoxic conditions. Mechanistically, OTUD6B directly interacts with pVHL and enhances its stability. In human HCC tissues, the protein level of OTUD6B was positively correlated with pVHL, whereas HIF-1α and vascular endothelial growth factor were negatively correlated. This study demonstrates that OTUD6B is a direct transcriptional target of HIF-1α, providing a new strategy for targeting hypoxic microenvironments for HCC therapy (48).

### ZRANB

ZRANB1 overexpression was associated with poorer survival in patients with HCC, and there was a significant positive correlation between the expressions of ZRANB1 and LOXL2 in clinical HCC specimens, which can regulate the expression of LOXL2 through specific protein 1 (SP1). Mechanistically, ZRANB1 stabilizes and binds SP1 through deubiquitination, which promotes liver cancer progression (17). However, another study reported that the deletion or downregulation of ZRANB1 was closely associated with the recurrence, metastasis, tumor volume, and disease stage of liver cancer significantly increased. Knockdown of ZRANB1 promotes HCC growth and metastasis by regulating Twist1 K63 ubiquitination (49).

## Ubiquitin-Specific Protease

The USP family is the most frequently studied DUB family and is a large family of more than 60 DUBs. The USP protein is considered an antagonist of the E3 ligase and is a potential target for cancer treatment (50). Here, we introduce the structure and function of “USP14, USP1, USP10, USP39, USP22, USP9X, USP2, USP7, USP4, USP5, USP29, USP15, USP12, USP16, USP27, USP46, and USP8” in the USP family research progress in HCC.

### USP14

#### Structure of USP14

The full length of the protein sequence of USP14 contains 494 amino acids. Its structure can be roughly divided into the N-terminal ubiquitination active center and C-terminal deubiquitination catalytic activity domain. The N-terminus has a 9-kDa ubiquitin-like (Ubl) domain, which is an important regulator of proteasome activity (51, 52); the C-terminus is a 45-kDa catalytic domain responsible for its DUB activity (53). The catalytic domain of USP14 is similar to the structure of the HAUSP catalytic core domain, which is an extended right hand consisting of three domains of fingers, palm, and thumb (51). When the apolipoprotein USP14 binds to the proteasome, the conformation of the two surface rings (BL1 and BL2) changes to bring the ubiquitin C-terminus into the catalytically active site (54, 55).

#### The Function of USP14 and Research Advances in Hepatocellular Carcinoma

Many studies have shown that USP14 can be involved in modulating a variety of signaling pathways associated with human diseases, such as cancer, autophagy, immune response, and viral infections (56, 57). In HCC, USP14 is highly expressed in liver cancer and is associated with a poor prognosis in patients with HCC. In the hypoxic environment of liver cancer (58), USP14 can enhance the transcriptional activity of HIF-1α and the stability of HIF-1α through deubiquitination, which in turn promotes the migration and invasion of HCC cells in a HIF-1α-dependent manner (59). This suggests that USP14 is a potential diagnostic biomarker for HCC as well as a therapeutic target. IU1, an inhibitor of USP14, can significantly inhibit the proliferation of liver cancer cells and liver cancer tissue tumors. It can be used as a potential HCC treatment agent *in vivo* and *in vitro*.

### USP1

#### Structure of USP1

USP1 regulates cellular DNA repair processes (60). USP1 has highly conserved USP domains of His and Cys and also has a catalytic triad consisting of C90, H593, and D751 (61). The protein sequence of USP1 consists of 785 amino acids, and the protein molecular weight is about 88.2 kDa (62). The cofactor UAF1 is a related factor of USP1 (63), which regulates the activity of USP1 isopeptidase by combining with UAF1 into a unique exogenous dimer complex. The enzyme activity of USP1 alone is low, but the activity of the enzyme is increased when combined with UAF1 (64, 65).

#### The Function and Research Progress of USP1 in Hepatocellular Carcinoma

In addition to being a regulator of cellular DNA repair (60), USP1 is also involved in the occurrence and development of various human diseases, such as USP1, plays a key role in the Fanconi anemia pathway (60), is a potential target for differentiation therapy (66), is upregulated in breast cancer, and is associated with poor patient prognosis (67). USP1 can also affect the development of lung cancer by regulating the PHLPP1-Akt signaling axis (68). In liver cancer, USP1 is thought to play a key role in the immune infiltration process of tumors. Drugs such as pimozide and ML-323 can inhibit the promotion of USP1 on the cell cycle and proliferation of HCC (69).

Ribosomal protein S16 (RPS16) is a highly conserved 40S ribosomal protein, which has been reported to be highly expressed in various cancers, such as colorectal cancer (CRC) (70). Studies have shown that USP1 can promote the stability of RPS16 protein and promote the proliferation and migration of liver cancer cells by binding to the cys90 (C90) site at the N-terminus of UAF1 (a cofactor of USP1) (71).

Protein transduction protein (TBL1) is a key regulator of the Wnt pathway and is proven to be associated with tumors in several studies, such as in cervical (72), prostate (73), and ovarian cancers (74). In liver cancer, USP1 can maintain the survival of hepatic circulating tumor cells by deubiquitinating and stabilizing TBL1 protein (75).

Lenvatinib (Lenvima) is an oral small-molecule inhibitor of multiple receptor tyrosine kinases for the treatment of advanced liver cancer patients (76). However, most patients will develop resistance to lenvatinib (77), so research on the mechanism of drug resistance in patients will help the development of targeted therapy for liver cancer (78). USP1 can promote the proliferation and migration of HCC cells by promoting the expression and stability of c-kit protein, and USP1 also promotes the efficacy of lenvatinib in HCC (79). In conclusion, USP1, as a novel diagnostic and predictive marker in the treatment of liver cancer, can provide new ideas for the development of targeted drugs for liver cancer treatment.

### USP10

#### Structure of USP10

USP10 is a cysteine protease of approximately 798 amino acids in length and is a highly conserved protein in eukaryotes (80). The catalytic domain of USP10 is located at 415 amino acids at the N-terminus of the protein and is about 380 amino acids in size. USP10 can remove Ub from the target protein by undergoing a hydrolysis reaction (80).

#### The Function of USP10 and Research Progress in Hepatocellular Carcinoma

USP10 is involved in many physiological activities in the human body, such as promoting cell proliferation and differentiation by targeting p53 protein (81); USP10 can activate the downstream protein AMPK through deubiquitination and form a feedforward loop with it (82). In addition, USP10 is also a tumor-related factor in human lung cancer (83), CRC (84), liver cancer, etc. (30). In HCC, multiple studies have shown that the transforming growth factor β (TGF-β) pathway is closely related to the metastasis of HCC (85, 86). USP10 can directly bind to Smad4 and act on the Lys-48-linked polyubiquitin chain on Smad4 to stabilize it; USP10 regulates the abundance and function of Smad4 protein through deubiquitination and activates the TGF-β pathway to further promote the migration of hepatoma cells (87). In addition, the USP10 inhibitor Spautin-1 can inhibit HCC metastasis in a dose-dependent manner, which makes it a targeted drug for effective anti-metastatic agents in the treatment of HCC.

mTOR signaling is highly expressed in liver cancer and other cancers (88). PTEN and AMPKα signaling pathways are regulators upstream of mTOR activation (89). USP10 acts as a tumor suppressor and acts as a tumor suppressor protein in HCC. USP10 stabilizes PTEN and AMPKα in HCC cells through deubiquitination and can inhibit AKT 329 phosphorylation and mTORC1 activation in HCC cells, thereby inhibiting the mTOR pathway (90).

A study showed that USP10 interacts with lncRNA GASAL1 to promote the malignancy of HCC (91). Mechanistic analysis revealed that lncRNA-GASAL1 could upregulate USP10 expression by targeting downstream miR-193b-5p through competitive binding. In addition, USP10 can stabilize proliferating cell nuclear antigen (PCNA) through deubiquitination to enhance the proliferation and migration of hepatoma cells (92).

YAP protein is a regulator found in *Drosophila* to control organ size (93, 94). Studies have shown that the Hippo-YAP/TAZ pathway is closely related to human metabolism, organ regeneration, and cancer (95–97). In HCC, USP10 was shown to activate YAP/TAZ protein and stabilize its activity through deubiquitination. USP10 can upregulate the abundance of YAP/TAZ protein in HCC and promote the proliferation and migration of HCC *in vivo* and *in vitro* (30). These provide new ideas and research proof for the mechanism of USP10 in HCC.

### USP39

#### Structure of USP39

Family member ubiquitin-specific peptidase 39 (USP39) is the homolog of Sad1p in yeast, also known as the human 65-kDa SR-related protein (98, 99). The structure of USP39 includes a central zinc finger ubiquitin domain and a canonical UCH domain (100). Studies have shown that there are no active site residues of cysteine and histidine in the structure of USP39, so there is no DUB enzyme activity (101), and it is also classified as a DUB (99).

#### The Function and Research Progress of USP39 in Hepatocellular Carcinoma

USP 39 (USP39) is an important regulator of human mRNA splicing and is highly expressed in a variety of cancers (100, 102). New research shows that USP39 plays a key role in the occurrence and development of liver cancer. The Kaplan–Meier analysis found that the high expression of USP39 in liver cancer was closely related to the poor prognosis of patients. USP39 may promote the malignancy of liver cancer by participating in the regulation of the epithelial–mesenchymal transition (EMT) pathway of HCC. ZEB1 is a key factor in the human tumor EMT pathway (103, 104). Mechanistic studies suggest that USP39 stabilizes ZEB1 protein through deubiquitination and activates the development of the EMT pathway and the proliferation and migration of hepatoma cells (105).

USP39 can directly bind and interact with the ubiquitinated E3 ligase TRIM26 (105). Studies have shown that the E3 ligase TRIM26 can inhibit the occurrence and development of several tumors in humans (106). USP39 and TRIM26 promote HCC progression through antagonism to balance the expression level of ZEB1 (105).

USP39 can be acetylated by the acetyltransferases HAT and MYST1. Acetylated USP39 can be degraded by E3 ubiquitin ligase (VHL)-mediated proteasome (107). SIRT7 has been reported to be an oncogenic potential factor in HCC and can form a regulatory loop with miRNAs to promote HCC progression (108). In the development of hepatoma cells, SIRT7 can deacetylate USP39, which improves the stability of USP39 and promotes the proliferation of HCC (107).

FoxM1 is widely recognized as a key factor in the transcriptional regulation of human cancers (109). It can promote the occurrence and development of HCC by regulating the expression of KIF4A (110). USP39 has been reported to promote the cleavage of forkhead box protein M1 (FoxM1) in hepatoma cells to promote the occurrence and development of HCC (111); USP39 knockdown can also induce apoptosis by targeting FoxM1 shear force on mRNA and promote the growth of hepatoma cell SMMC-7721 *in vitro* and *in vivo* (112).

Specific protein 1 (SP1) belongs to the Sp/KLF transcription factor family (113) and is considered to be the basal transcription factor in humans. Sp1 is also associated with a variety of human diseases, such as Huntington’s disease (113, 114). SP1 is also associated with poor prognosis in a variety of cancers (115). Studies have shown that USP39 can stabilize Sp1 and prolong its half-life through deubiquitination in HCC (116). In addition, USP39 can also promote the SP1-dependent pathway. Therefore, USP39 can target Sp1 to promote liver cancer cell proliferation (116).

### USP22

#### Structure of USP22

USP 22 (USP22) is an important member of the USP family. Its protein structure consists of 525 amino acids, including the structural sequence of a putative ubiquitin hydrolase containing a C-terminal peptidase domain and an N-terminal UBP-type zinc finger motif (117). In addition, USP22, ATXN7L3, ATXN7, and ENY2 are transcriptional cofactors of human Spt-Ada-Gcn5 acetyltransferase (hSAGA) and key subunits of the SAGA complex (118, 119).

#### The Function and Research Progress of USP22 in Hepatocellular Carcinoma

As an important member of the USP family, USP22 also plays a very important role in the occurrence and development of HCC. Among them, the expression of USP22 and survivin was shown to be closely related to the malignant behavior of HCC cases, including tumor size, stage, and differentiation (120). Several studies have reported that USP22 is closely related to the drug resistance mechanism of HCC. For example, in sorafenib-resistant cell lines, USP22 can regulate and upregulate ABCC1 (121). In addition, USP22 can directly interact with SIRT1 and regulate the protein expression level of SIRT1, which promotes the resistance of hepatoma cells to 5-fluorouracil (5-FU) (122). Previous reports have demonstrated that SIRT1 can deacetylate and activate the AKT pathway (123), and USP22 can promote MDR in HCC cells by activating the SIRT1/AKT/MRP1 pathway (124); USP22 is also able to regulate chemotolerance in HCC through Smad4/Akt-dependent MDR-related gene regulation (117). Relevant drug resistance genes include BMI1 and EZH2. Co-expression of USP22 and BMI1 is associated with poor prognosis and enhanced anticancer drug resistance in HCC (125). Some researchers have proposed a self-activating cascade reaction—the co-delivery system of sorafenib and shUSP22 (Gal-SLP), aiming at the effect of USP22 on the drug resistance of liver cancer cells. This delivery system exhibits potent antitumor efficiency through three synergistic effects (126). This is also a major advance in the use of DUBs for the treatment of HCC. We presume that with the in-depth study of DUB, DUBs can provide new approaches and strategies for the treatment of cancer in humans. In addition to affecting the drug resistance of liver cancer cells, USP22 can also regulate peroxisome proliferator-activated receptor γ (PPARγ) in HCC through deubiquitination to promote fatty acid synthesis and tumorigenesis. These findings provide a new therapeutic strategy for patients with high USP22 expression in HCC (127).

In addition to the effect on drug resistance of liver cancer cells, other studies have also reported that USP22 can significantly affect the glycolysis and stemness characteristics of liver cancer cells under hypoxic conditions: HIF-1α knockdown inhibits USP22-induced and hypoxia-induced effects (128). USP22 can also affect the transcription of the phosphatase DUSP1 by E2F6 protein through deubiquitination, which can activate the AKT pathway in hepatoma cells (129). In addition, USP22 can also be regulated by lncRNA HULC to further affect the drug resistance and tumor growth of liver cancer cells (130, 131).

### Other Enzymes of the USP Family

In addition to the abovementioned USP family DUBs, there are other liver cancer-related USP family DUBs, including USP9X, USP2, USP7, USP4, USP5, USP29, USP15, USP12, USP16, USP27, USP46, and USP8.

#### Their Function and Research Progress in Hepatocellular Carcinoma

##### USP9X

USP9X has been proved by many studies to affect the occurrence and development of HCC (132). For example, by promoting HCC cell proliferation by regulating the expression of β-catenin (eta-catenin) (133), USP9X is able to affect hepatoma cells with ARID1A mutations through the AMPK pathway (134), miR-26b can regulate USP9X-mediated p53 deubiquitination to enhance the sensitivity of HCC cells to doxorubicin (135), miR-26b is also able to target USP9X expression to suppress EMT in hepatocytes (136), and usp9x can affect the drug sensitivity of hepatoma cells to doxorubicin and WP1130 through p53 (137). The lncRNA LINC00473 is also able to exert its oncogenic function in HCC by interacting with USP9X and may be a therapeutic target for HCC treatment (138).

#### USP2

USP2a is significantly upregulated in HCC tissues and positively correlated with poor patient prognosis, and USP2a can promote HCC progression by deubiquitinating and stabilizing RAB1A (139). In addition, USP2a is also believed to be involved in the production of nascent adipose to further regulate the progression of HCC, which has pathogenic and prognostic significance for HCC (140). USP2b has been shown to be dysregulated in HCC patients, promoting apoptosis and necrosis of HepG2 and Huh 7 cells. This study demonstrates that USP2 contributes to the pathogenesis of HCC and provides a molecular basis for the development of HCC therapies by modulating USP2b expression or activity (141).

#### USP7

The expression of USP7 is significantly increased in HCC and has been reported to have clinical significance in the prognosis and functional mechanism of HCC (142). USP7 may be a drug target for chemoresistance in HCC (143). MicroRNA-205 (miR-205) may negatively regulate the UPS7 protein level by targeting the 3′-untranslated region in HCC cells (144). Adipocyte-secreted exosomal circRNAs promote tumor growth and reduce DNA damage by inhibiting miR-34a and activating USP7/Cyclin A2 signaling pathway (145). METTL3 can regulate the expression of USP7 through m6A methylation and promote the invasion, migration, and proliferation of HCC cells (146). Furthermore, the homolog of Usp7, HAUSP, is able to regulate the Hippo pathway and stabilize Yorkie (Yki) and HAUSP as potential therapeutic targets for HCC (147). USP7 can also bind to FEN1, a poor prognostic molecule in HCC through deubiquitination, which can reduce the expression of p53 and promote the progression of HCC (148). In liver cancer, PROX1 can also enhance the stability of p65 by binding USP7 to affect angiogenesis in liver cancer cells (149). USP7 promotes HCC cell growth by forming a complex with thyroid hormone receptor-interacting protein 12 (TRIP12) and stabilizing p14 (ARF) ubiquitination, thereby promoting HCC progression (150).

##### USP4

Kaplan-Meier survival analysis showed that patients whose tumors overexpressed USP4 had poor overall survival, and it combined with cyclophilin A (CypA) to form a complex to activate the MAPK signaling pathway in HCC (151). In addition, USP4 can directly interact with TGF-β receptor type I (TGFR-1) through deubiquitination and activate the TGF-β signaling pathway, which can induce EMT in hepatoma cells, providing a new therapeutic target for the treatment of HCC (152). USP4 is able to act as a downstream target of miR-148a in hepatoma cells, and overexpression may contribute to the progression of HCC to more aggressive features (153).

##### Others

USP5 has been reported to be highly expressed in human hepatoma cells and can inhibit the expression of p53 and DNA repair function (154). It also binds to SLUG and regulates the EMT pathway associated with hepatoma cells (155).

The expression of USP13 was significantly upregulated in HCC cells, and studies showed that USP13 knockdown could inhibit the activation of the TLR4/MyD88/NF-κB pathway in hypoxia-induced HCC cells. In addition, studies have shown that USP13 can affect the growth of liver cancer cells by regulating the expression of c-Myc (156, 157).

Studies have shown that USP29 is related to HIF-1α in hepatoma cells. Mechanistically, USP29 promotes sorafenib resistance in HCC cells by upregulating glycolysis, thus opening a new avenue for therapeutic targeting of patients with sorafenib-resistant HCC (158).

USP15 is highly expressed in liver cancer tissues and cell lines, and high expression is significantly positively correlated with HCC recurrence. Studies have shown that downregulation of USP15 expression can inhibit the proliferation and apoptosis of liver cancer cells (159). In addition, xanthine oxidoreductase (XOR) can interact with USP15 to enhance the stability of Kelch-like ECH-associated protein 1 (KEAP1), which ultimately promotes the accumulation of reactive oxygen species (ROS) and liver cancer stem cells (CSCs) (160).

USP12 promotes HCC proliferation and apoptosis by affecting p38 and MAPK pathways (161). USP16 is downregulated in HCC, leading to Ct-HBx promoting the tumorigenicity and malignancy of HCC (162). USP27 promotes its stability by interacting with SETD3 and accelerates the growth of hepatoma tumor cells, and higher expression of USP27 and SETD3 predicts poorer survival in HCC patients (163). USP46 can promote MST1 kinase activity through deubiquitination to inhibit tumor growth and metastasis, suggesting that USP46 may be a potential therapeutic strategy for HCC (164). USP8 can regulate the expression of multiple receptor tyrosine kinases (RTKs) to affect the drug resistance of liver cancer cells (165).

## Ubiquitin C-Terminal Hydrolase

The family of UCHs includes UCH-L1, UCH-L3, UCHL5/UCH37, and BRCA1-associated protein-1 (BAP1) (166). The UCH family has a classically conserved catalytic domain of about 230 amino acids in size (167). The domains of UCH-L5, UCH-L1, and UCH-L3 contain an active site crossover loop (116, 166). UCHL1 has been reported to be strongly associated with Parkinson’s disease (PD) (168, 169) and Alzheimer’s disease (AD) in humans (170). In view of the lack of current research reports on the UCH family, here we only introduce the structure and function of UCH37 and the research progress in HCC.

### UCH37

#### Structure of UCH37

UCH37, also known as UCHL5, belongs to the human UCH family and is the only DUB in the family that is associated with the mammalian proteasome (171, 172). The protein structural sequence of the protease UCH37 contains 329 amino acids and is mainly associated with the Ub isopeptidase activity in the 19S proteasome regulatory complex (173). It is also the only UCH family of proteases capable of acting on the 19S proteasome complex and cleaving Lys48-linked polyubiquitin molecules in a unique manner (174). The three-dimensional structure of UCH37 consists of two parts, a globular UCH domain and a fibrillar unique C-terminal extension (175). Studies have shown that NFRKB can inhibit its activity by interacting with the extended structure of the C-terminus of UCH37 (173). During deubiquitination, UCH37 is able to associate with the 26S proteasome *via* Rpn13.

#### The Function and Research Progress of UCH37 in Hepatocellular Carcinoma

Studies have shown that UCH37 is highly expressed in liver cancer cells (HCC) and cancer tissues, and the prognosis of patients is poor (176). Peroxiredoxin 1 (Prdx1) belongs to the peroxidase family and plays a dual role in human tumorigenesis (177). Multiple studies have shown that Prdx1 is involved in the progression of human liver cancer, including tumor angiogenesis (178), apoptosis, autophagy (179), and poor patient prognosis in HCC (180). PRDX1 low expression can promote the proliferation, migration, and invasion of HCC cells *in vitro*. New research shows that the interaction of Prdx1 with UCH37 attenuates the effects of UCH37 on cell migration and invasion; this interaction may be through the formation of a complex rather than the deubiquitination of UCH37 itself, but the mechanism of the two on the development of liver cancer has not yet been elucidated (181). UCH37 can also act on the RNA splicing factor PRP19 through deubiquitination (182), and their interaction can promote HCC migration and invasion (176).

The protein chaperone GRP78 is often highly expressed in human cancers (183), such as lung cancer (184), pancreatic cancer (185), and breast cancer (186). GRP78 is also associated with diseases such as tumor resistance, patient prognosis (187), M2 macrophage polarization (188), and folding of nervous system proteins (189). The latest study shows that UCH37 can interact with the protein chaperone GRP78 by co-immunoprecipitation and confocal laser scanning microscopy, which provides new ideas and directions for the mechanism of UCH37 in HCC (190).

## DUB-Related Inhibitors

As we know, proteasome inhibitors have been developed and used successfully in the treatment of some diseases (191, 192), which lays the foundation for the development of DUB as a drug research target. Currently reported inhibitors such as PR-619 and WP1130 can inhibit a variety of DUBs, of which WP1130 inhibits at least five DUBs: USP5, UCH-L1, USP9X, USP14, and UCH37 (193). However, the development of specific inhibitors has been challenging, which is related to the highly conserved structural features of the DUB catalytic site.

In the DUB family, UPS has been clearly regarded as one of the most important drug targets, and the research and development of inhibitors are more in-depth than those of other families. Among them, USP14 has been researched and developed as a more mature inhibitor. The research group of Finley et al. identified more than two hundred inhibitors of USP14 based on high-throughput screening of Ub-AMC hydrolysis assays; IU1 is the first specific inhibitor targeting USP14, named IU1 (194). In addition, other inhibitors have been developed: IU1 analogs, such as IU1-206 and IU1-248 and IU2 series (195); spautin-1 is a small molecule inhibitor of USP10 and also inhibits USP13 (196). Recent research reports identify Wu-5 as a novel USP10 inhibitor that induces degradation of the FLT3 mutant protein (197). A ubiquitin variant (UbV) phage library has also been used to develop an inhibitor-UbV.7.2 that can target USP7 and USP10, which structurally enhances the affinity for USP7 (198). A leukemia drug, 6-thioguanine, was found to be a potent inhibitor of USP2, exhibiting a non-competitive and slow-binding inhibitory mechanism for USP2 (199). Studies have reported that inhibitors of USP8 include RA-9, DUB-IN-1, DUBs-IN-2, and a novel inhibitor DC-U43 (200–202). Among them, DC-U43-10 is a USP8 inhibitor with a novel scaffold, which can bring new research directions for the development and clinical research of USP8 inhibitors (203). Morgan et al. screened a large number of cyclic peptide combinatorial libraries and identified the first inhibitors of USP22, which have broad prospects for development (204). Furthermore, WP1130 is a general inhibitor. UCH37-specific inhibitors have not yet been developed, but there are some non-specific DUB inhibitors targeting UCH37 activity, such as b-AP15, Ub-AMC, Ub-Rho, and WP1130 (195). Although UCH37 lacks specific inhibitors, the developed multi-target inhibitors can also provide new strategies and ideas for clinical drug development. We summarize some inhibitors of DUB in **Table 1**.

**Table 1:** DUB-related targets, inhibitors, and pathways in HCC and corresponding articles.

DUB		Downstream pathway, target protein	Mechanism of action in HCC	Article	Inhibitors
OTU	OTUB1	MIR-493-5P/OTUB1/FOXO3a	Metastasis, proliferation, invasion,	(38)	\
	OTUD3	OTUD3/ACTN4	Metastasis, proliferation	(47)	\
	OTU6B	HIF-1α/OTU6B/PVHL	Promote HCC growth	(48)	\
	ZRANB1	ZRANB1/SP1/LOXL2	Promote HCC growth	(17)	\
		ZRANB1/Twist1	Growth, metastasis	(49)	
USP	USP14	USP14/HIF-1α	Metastasis, invasion, apoptosis	(59)	IU1, IU2, SB1-B-57
	USP1	USP1/TBL1	Promote HCC growth	(75)	GW7674, ML323, 1-173
		USP1/C-Kit	Metastasis, proliferation, drug resistance	(79)	
	USP10	USP10/Smad4	Metastasis Activate TGF-β pathway	(87)	Spautin-1, Wu-5
		USP10/PTEN, AMPKα	Activate M-TOR pathway	(89)	
		GASAL1/MIR-193b-5p/USP10/PCNA	Promote HCC growth	(92)	
		USP10/YAP/TAZ	Metastasis, proliferation	(30)	
	USP39	USP39/ZEB1/AKT	Metastasis, proliferation	(105)	\
		USP39/FOXM1	Apoptosis	(111)	
		SIRT7/USP39	Proliferation	(107)	
		USP39/SP1	Proliferation	(116)	
USP	USP22	USP22/ABCC1	Drug resistance	(121)	Macrocyclic
		USP22/SIRT1	AKT/drug resistance	(122)	
		USP22/SMAD4	AKT/promote MDR	(117)	
		USP22/BMI1	Promote MDR	(125)	
		USP22/HULC	Drug resistance	(130, 131)	
		HIF-1α/USP22	Cell stemness, glycolysis	(128)	
		USP22/E2F6/DUSP1	AKT	(129)	WP1130, degrasyn
	USP9X	USP9X/β-Catenin	Proliferation	(133)	
		MIR-26b/USP9X/P53	EMT/drug resistance	(136)	
		USP9X/LINC00473	Promote HCC growth	(138)	
	USP2	USP2A/RAB1A	Promote HCC growth	(139)	STD1T, 6TG, Q29, ML364, LCAHA
	USP7	MIR-205/USP7	Promote HCC growth	(144)	Ursolic acid, GNE6640, XL188, FT671, ALM2, 1-8
		CircRNA/MIR-34a/USP7	Reduce DNA damage	(145)	
		METTL3/USP7	Metastasis, proliferation, invasion	(146)	
		USP7/FEN1/P53	Promote HCC growth	(148)	
		USP7/RPOX1/P65	Angiogenesis	(149)	
		USP7/TR2P12/P14	Promote HCC growth	(150)	
	USP4	USP4/CYPA	Activate MAPK pathway	(151)	Degrasyn
		USP4/TGFR-1	Activate TGF-β pathway	(152)	
		MIR-148a/USP4	Metastasis, invasion,	(153)	
	USP5	USP5/P53	DNA repair,	(154)	Degrasyn, Vialinin A
		USP5/SLUG	Activate EMT pathway	(155)	
	USP13	USP13/C-MYC	Promote HCC growth	(156)	Spautin-1
		USP13/TLR4/MYD88	Activate NF-kB pathway	(157)	
	USP29	HIF-1α/USP29	Glycolysis, drug resistance	(158)	WP1130
	USP15	USP15/XOR/KEAP1	Cell stemness, ROS	(159)	WP1130
	USP12	USP12/P38	MAPK Proliferation, apoptosis,	(161)	WP1130
	USP16	USP16/CT-HBX	Promote HCC growth	(162)	WP1130
	USP27	USP27/SETD3	Promote HCC growth	(163)	WP1130
	USP46	USP46/MST1	Growth, metastasis	(164)	WP1130
	USP8	USP8/RTKS	Drug resistance	(165)	HY50737A HY0736 DC-U43-10
UCH	UCH37	UCH37/PRDX1	Metastasis, invasion,	(181)	b-AP15, Ub-AMC, Ub-Rho
		UCH37/GRP78	\	(190)	

HCC, hepatocellular carcinoma; MDR, multidrug resistance; ROS, reactive oxygen species.

## Summary and Outlook

Based on the introduction and analysis of some liver cancer-related DUBs in this paper, it can be seen that DUBs play a unique regulatory role in the occurrence and development of HCC.

However, the regulatory mechanism of DUBs in liver cancer is relatively complex, involving many pathways and targets, and the development of targeted drugs has become an important treatment method for patients with high DUB expression in HCC. At present, molecularly targeted drugs and small molecule inhibitors for ubiquitination and deubiquitination-related enzymes have been used in the clinical treatment of cancer (205, 206). Drugs such as oprozomib, ixazomib, and bortezomib have achieved remarkable therapeutic results (207). In the article, we also summarize some inhibitors of liver cancer-related DUBs. The current research results show that the USP family-related inhibitors are widely studied. Represented by USP14, the research on IU1 is relatively mature and has great potential for clinical application. USP22, USP14, USP10, USP13, USP7, USP2, and USP8, liver cancer-related DUBs, have also been reported to have related small molecule inhibitors, but the research and development are not mature enough. Using a large library of cyclic peptides in high-throughput screening, researchers recently identified the first inhibitors of USP22—macrocyclic inhibitors. In addition, UCH37 of the UCH family also has small molecule inhibitors, but these are not specific inhibitors of UCH37. Due to the unique active structure of DUB, there are still some difficulties in the development of many small molecule inhibitors. In conclusion, the prospect of DUB inhibitors as drug targets is still very impressive, and it will also have great clinical application significance for the treatment of human diseases in the future.

In this article, we introduce that OTUB1 OTUD3 OTUD6B ZRANB1 USP14, USP1, USP10, USP39, USP22, USP9X, USP2, USP7, USP4, USP5, USP29, USP15, USP12, USP16, USP27, USP46, USP8, and UCH37 can affect the malignant degree of HCC through the corresponding mechanism. Among them, USP22, USP1, and USP9X were all related to drug resistance to HCC; USP14, USP13, USP29, and OTU6B were all related to the hypoxic microenvironment and HIF in HCC. As can be seen, the relationship between the USP family and liver cancer is currently the most frequently studied with the participation of a class of DUBs, and many corresponding small molecule inhibitors have also been studied for such DUBs. Therefore, we presume that the USP family is the most promising biomarker for DUB for the diagnosis and treatment of liver cancer. Among them, USP14 small molecule inhibitors are the most clinically significant drug targets. However, the research progress on OTU and UCH family in liver cancer is less. There are also very few reports on Machado–Joseph domain-containing proteases (MINDY) and zinc-dependent metalloproteinases (JAMMs), so the research on the mechanism of DUBs in liver cancer is far from being in-depth. There is still a lot of room for development in the study of DUBs on the pathogenesis and treatment of liver cancer. The development of related targeted drugs and the clinical application of small molecule inhibitors will also become a research hotspot in the future. These can provide new ideas and research directions for the treatment of liver cancer in the future.

## Author Contributions

(I) Conception and design: JieZ, JG. (II) Administrative support: QX, XH. (III) Collection and assembly of data: YW, QM, YS, QL, WF, FC, GO, JiZ. All authors contributed to this article and agree to the submission of the article version.

## Conflict of Interest

The authors declare that the research was conducted in the absence of any commercial or financial relationships that could be construed as a potential conflict of interest.

## Publisher’s Note

All claims expressed in this article are solely those of the authors and do not necessarily represent those of their affiliated organizations, or those of the publisher, the editors and the reviewers. Any product that may be evaluated in this article, or claim that may be made by its manufacturer, is not guaranteed or endorsed by the publisher.
